# Supplementary material for: Isolation by distance promotes strain diversification in the wild mouse gut microbiota
Source: bioRxiv. 2025 Sep 17:2025.09.15.676373. Preprint. [Version 2] doi: 10.1101/2025.09.15.676373 (PMC12458340; doi:10.1101/2025.09.15.676373)
Supplement: Supplement 6 [file NIHPP2025.09.15.676373v2-supplement-6.pdf]

bioRxiv preprint doi: https://doi.org/10.1101/2025.09.15.676373; this version posted September 17, 2025. The copyright holder for this preprint (which was not certified by peer review) is the author/funder, who has granted bioRxiv a license to display the preprint in perpetuity. It is made available under aCC-BY 4.0 International license.

Supplementary Materials

Supplementary Figures

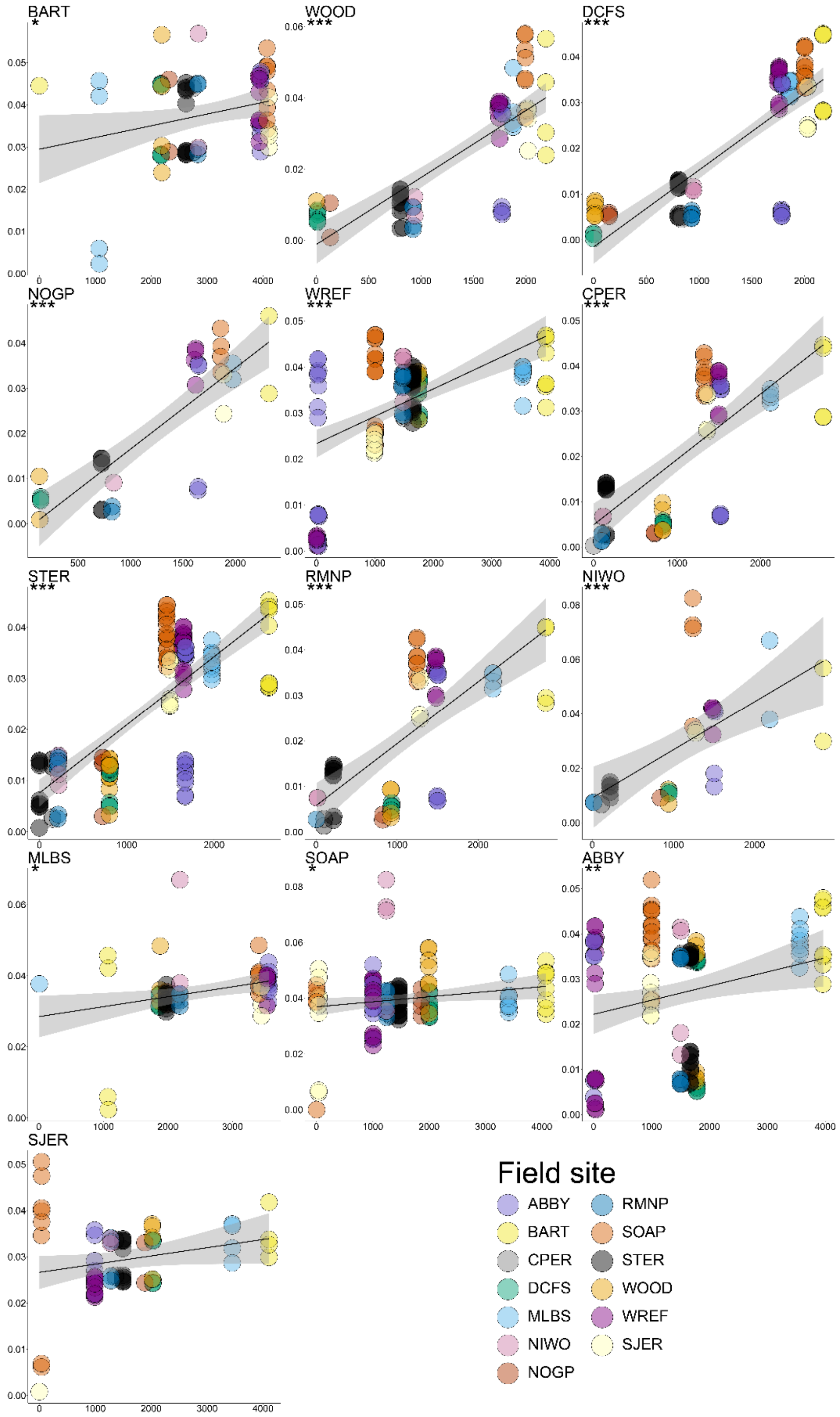

Fig. S1. Consistent evidence for IBD in deer mice across field sites. Scatter plots show IBD of deer mouse MT lineages across field sites. MT genetic distances and geographic distances are shown on the y- and x-axes, respectively. Each facet corresponds to a focal field site for which comparisons including that field site are shown. Points represent pairs of deer mice, with color denoting the non-focal field site. Trendline and shading indicate best-fit linear regression with standard error.

bioRxiv preprint doi: https://doi.org/10.1101/2025.09.15.676373; this version posted September 17, 2025. The copyright holder for this preprint (which was not certified by peer review) is the author/funder, who has granted bioRxiv a license to display the preprint in perpetuity. It is made available under aCC-BY 4.0 International license.

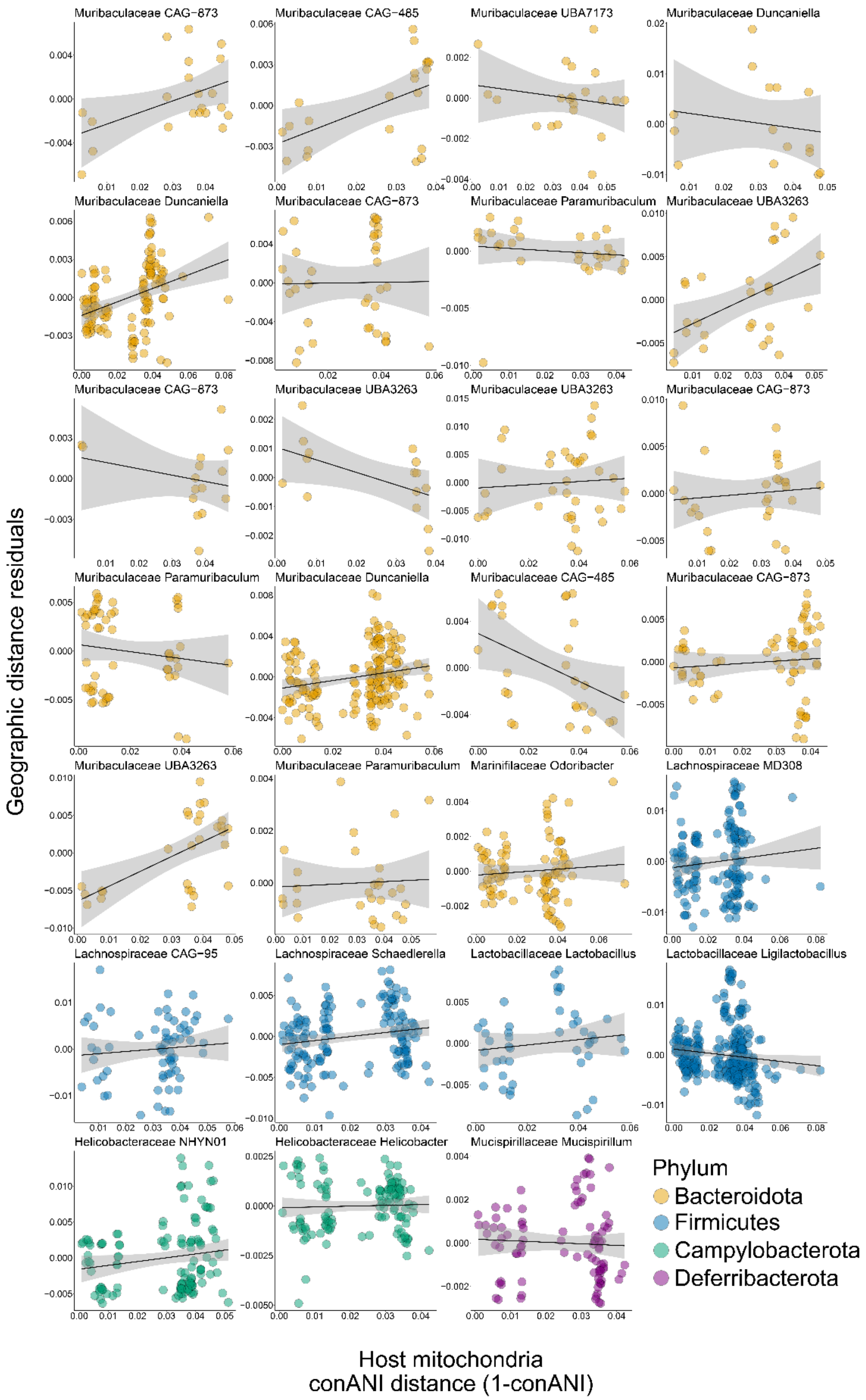

Fig. S2. No effect of IBH on strain diversification within gut bacterial species. Scatterplots and trendlines show lack of relationships between SGB conANI distances (residualized against geographic distances) and host MT distances for individual SGBs derived from deer mouse hosts. SGB ANI distances were residualized against the residuals from a GLS model with the formula: SGB ANI distance ~ geographic distance. Each facet represents an SGB. Colors denote the phylum to which each SGB belongs, as indicated by the key. Shading around each trendline shows standard error from the best-fit linear model. No SGBs reached significance at the MRM FDR-corrected p-values < 0.05 threshold.

bioRxiv preprint doi: https://doi.org/10.1101/2025.09.15.676373; this version posted September 17, 2025. The copyright holder for this preprint (which was not certified by peer review) is the author/funder, who has granted bioRxiv a license to display the preprint in perpetuity. It is made available under aCC-BY 4.0 International license.

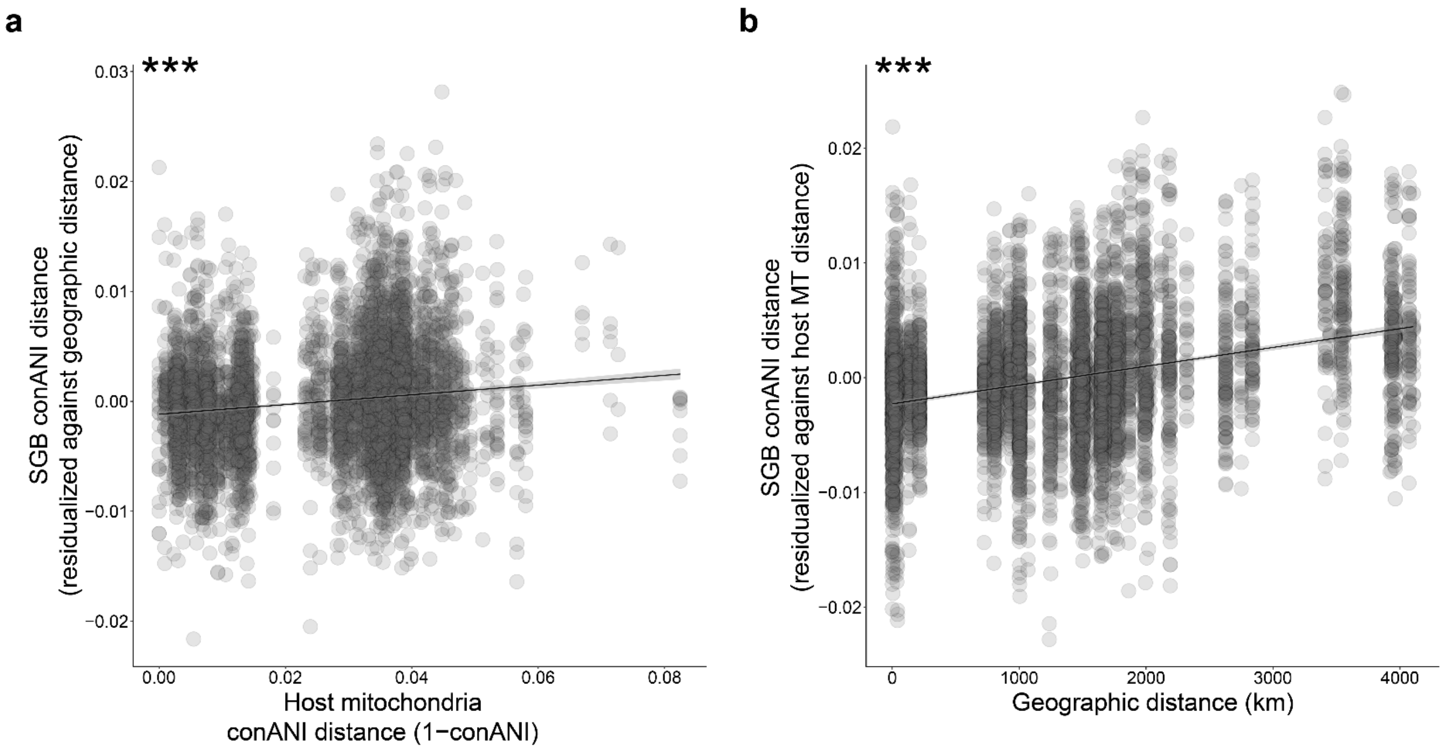

Fig. S3. Aggregating gut bacterial strain comparisons across SGBs indicates widespread IBD and relatively weaker IBH. A) Scatter plot shows the relationship between strain genomic similarity within SGBs (1 – conANI) and geographic distance, after accounting for the effects of host MT genetic distance. Y-axis shows genomic similarity (1 – conANI) residualized against host MT genetic distances. B) Scatter plot shows the relationship between strain genomic similarity within SGBs (1 – conANI) and host MT genetic distance, after accounting for the effects of geographic distance. Y-axis shows genomic similarity (1 – conANI) residualized against geographic distances. In (A) and (B), trendlines and shading indicate best-fit linear regressions with standard errors. Asterisks indicate regression p-value; *** < 0.001.

bioRxiv preprint doi: https://doi.org/10.1101/2025.09.15.676373; this version posted September 17, 2025. The copyright holder for this preprint (which was not certified by peer review) is the author/funder, who has granted bioRxiv a license to display the preprint in perpetuity. It is made available under aCC-BY 4.0 International license.

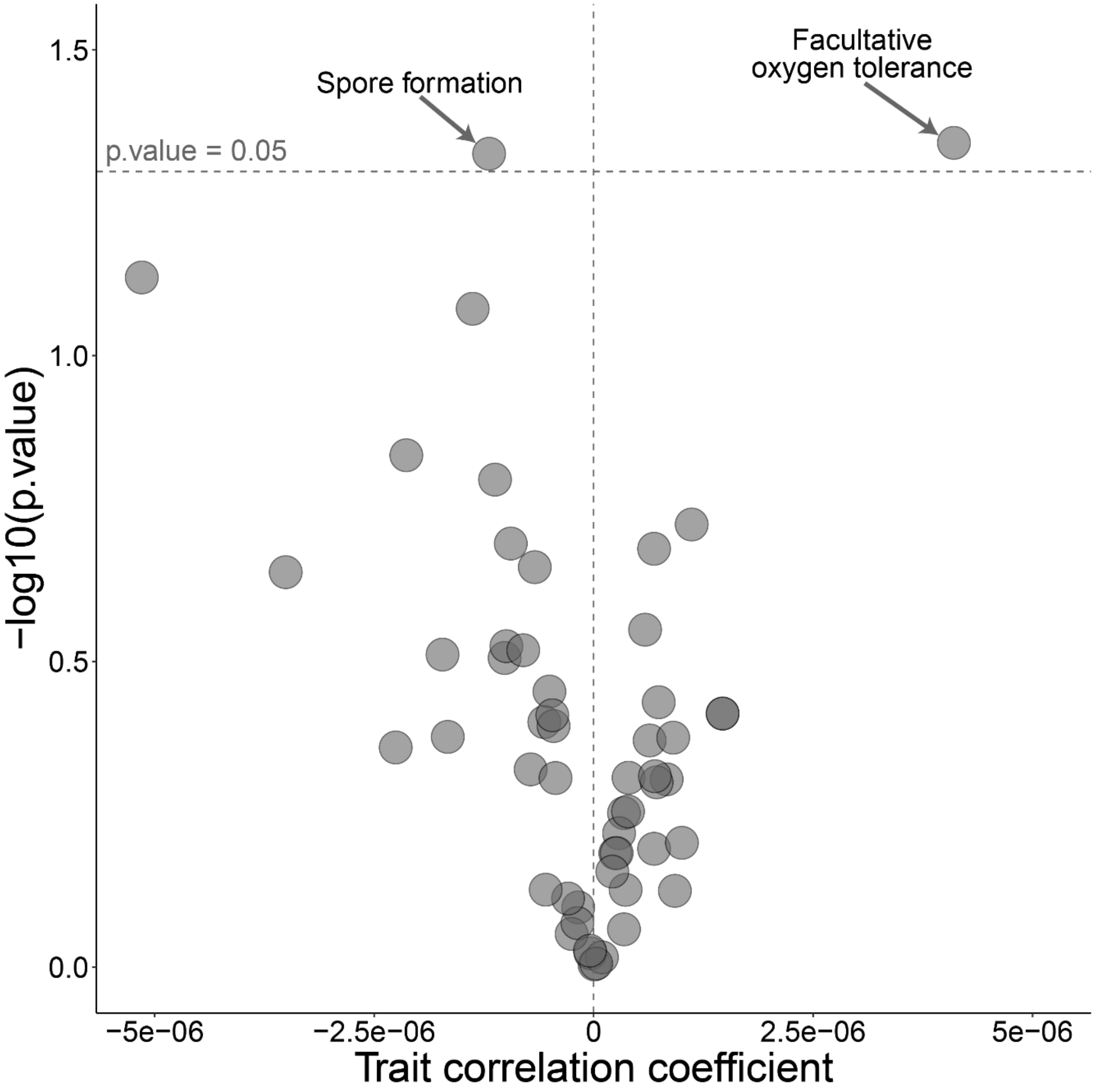

Fig. S4. Traitar analyses confirm negative relationship between sporulation ability and strength of IBD. Volcano plot shows the significance (y-axis) of association between the presence of bacterial traits (points) and the strength of IBD. The strength of IBD was measured as the MRM Pearson correlation coefficient describing the relationship between geographic distance and strain genomic divergence between mice within SGBs residualized against host MT genetic distances.

Supplementary Tables

Supplementary Table 1. Metadata for rodent fecal samples sequenced with Illumina NovaSeq.

Supplementary Table 2. Metadata and quality reports for metagenome-assembled genomes.

Supplementary Table 3. Results from MRM Pearson, MRM Spearman, and Residual MRM analyses.

Supplementary Table 4. Results from functional analyses.

Supplementary Table 5. Results from SGB genetic distance analyses in sympatric hosts.
